# Identification and Expression Profile Analysis of Odorant Binding Proteins in the Oriental Fruit Fly *Bactrocera dorsalis*

**DOI:** 10.3390/ijms140714936

**Published:** 2013-07-17

**Authors:** Weiwei Zheng, Wei Peng, Chipan Zhu, Qun Zhang, Giuseppe Saccone, Hongyu Zhang

**Affiliations:** 1 State Key Laboratory of Agricultural Microbiology, Hubei Key Laboratory of Insect Resource Application and Sustainable Pest Control and Institute of Urban and Horticultural Pests, College of Plant Science and Technology, Huazhong Agricultural University, Wuhan 430070, Hubei, China; E-Mails: zijing029@126.com (W.Z.); pengweijack@163.com (W.P.); wss1203@webmail.hzau.edu.cn (C.Z.); 15071238373@163.com (Q.Z.); 2 Department of Biological Sciences, University Federico II of Naples, Napoli 80138, Italy; E-Mail: giuseppe.saccone@unina.it

**Keywords:** odorant binding protein, olfaction, tissue expression pattern, *Bactrocera dorsalis*

## Abstract

Olfaction is crucial in many insects for critical behaviors, including those regulating survival and reproduction. Insect odorant-binding proteins (OBPs) function in the first step of the olfactory system and play an essential role in the perception of odorants, such as pheromones and host chemicals. The oriental fruit fly, *Bactrocera dorsalis*, is a destructive fruit-eating pest, due to its wide host range of up to 250 different types of fruits and vegetables, and this fly causes severe economic damage to the fruit and vegetable industry. However, *OBP* genes have not been largely identified in *B. dorsalis*. Based on our previously constructed *B. dorsalis* cDNA library, ten *OBP* genes were identified in *B. dorsalis* for the first time. A phylogenetic tree was generated to show the relationships among the 10 OBPs of *B. dorsalis* to OBP sequences of two other Dipteran species, including *Drosophila melanogaster* and the mosquito *Anopheles gambiae.* The expression profiles of the ten *OBPs* in different tissues (heads, thoraxes, abdomens, legs, wings, male antennae and female antenna) of the mated adults were analyzed by real-time PCR. The results showed that nine of them are highly expressed in the antenna of both sexes, except *BdorOBP7*. Four *OBPs* (*BdorOBP1*, *BdorOBP4*, *BdorOBP8*, and *BdorOBP10*) are also enriched in the abdomen, and *BdorOBP7* is specifically expressed in leg, indicating that it may function in other biological processes. This work will provide insight into the roles of OBPs in chemoreception and help develop new pest-control strategies.

## 1. Introduction

Olfaction is essential for insects in the location of hosts and the detection of pheromones during the recognition and location of mates. The olfactory system in insects consists of three main types of proteins: odorant binding proteins (OBPs), odorant receptors (ORs) and odorant-degrading esterases (ODEs) [1]. Of these proteins, OBPs often function in the first step of odor perception.

Insect OBPs are water-soluble proteins that often consist of 120–150 amino acids present in the chemosensory organs of insects. These proteins are able to bind various hydrophobic odorant molecules in the environment and transport them through the hemolymph to the ORs at the dendrite membrane of the olfactory sensory neurons with antennae. Then the olfactory signal transduction system is induced [2–4]. OBPs often contain six highly conversed cysteine residues, which have been used for its genome-wide identification and annotation in a range of insect species [5–9]. Based on the number of cysteine residues they contain, OBPs are now classified as “Classic”, “Minus-C”, “Plus-C”, “Dimer”, and “Atypical” OBPs [7,10,11].

Substantial evidence has shown that OBPs are critical in odorant recognition, rather than merely serving as passive odorant shuttles [12–18]. Response to the odorants ethanol or benzaldehyde was damaged in *Drosophila* that lacked the “LUSH” OBP [12,13]. Additionally, it has been reported that *Drosophila lush* mutants have a complete loss of sensitivity to the pheromone 11-*cis* vaccenyl acetate and the “LUSH” OBP is required for activity of pheromone-sensitive neurons [14]. Knock down of OBP1 in mosquitoes *Culex quinquefasciatus* reduced antennal response to several oviposition attractants, as measured by electrophysiological analyses [16]. *A. gambiae* OBP1 was demonstrated by RNAi to play an essential role in mediating indole recognition in the antennae of female mosquitoes [17]. Additionally, it has been demonstrated that different OBPs display distinct odorant-binding specificity in a single moth species [15,19,20]. All of these studies revealed that OBPs play significant roles in insect olfactory systems. Therefore, it is of great importance in the study of OBPs to understand the molecular basis of olfaction in insects and to develop environmentally friendly strategies for pest control.

Due to genome annotation and transcriptome sequencing, OBPs have been widely identified in many insect species, including the Dipteran species *Drosophila melanogaster* [7,10], the onion fly *Delia antiqua* [21], *Aedes aegypti* [8], *Culex pipiens quinquefasciatus* [22], *Anopheles funestus* [23], Lepidopteran *Bombyx mori* [24], *Manduca sexta* [25], Hymenopteran *Solenopsis invicta* [26], and Hemipteran *Adelphocoris lineolatus* [27]. However, OBPs have not been largely reported in Tephritid pest species that are of major economic importance in agriculture, except several partial sequences of the Mediterranean fruit fly, *Ceratitis capitata* [28].

The oriental fruit fly, *B. dorsalis* (Diptera: Tephritidae), is a highly invasive agricultural pest in Asian countries. Due to its wide host range of up to 250 different types of fruits and vegetables, this species causes severe economic losses every year. However, at present, there is only one cDNA sequence of *B. dorsalis OBP* in Genbank (accession number: EU564816). Therefore, it is of great importance to identify more OBPs that may be involved in host location and ovipositing in *B. dorsalis*.

In the present study, ten OBPs were identified from the *B. dorsalis* cDNA library we had previously constructed [29]. Sequence alignment and phylogenetic tree analysis was performed to characterize these molecules. A tissue distribution expression pattern of the mated adults was then inferred by quantitative RT-PCR. This work presents for the first time a study of the OBPs of the invasive agricultural pest *B. dorsalis*, which may provide more molecular targets for *B. dorsalis* control and insight into insect olfaction research, thus providing an essential foundation for the development of efficient, simple, green and sustainable pest control strategies.

## 2. Results and Discussion

### 2.1. Identification of OBPs in *B. dorsalis*

Nearly 20 OBPs were identified from our previously constructed *B. dorsalis* transcriptome [29], but only the full-length cDNAs encoding *B. dorsalis* OBPs were presented in this study (Table 1). These 10 OBPs all contain one conserved PBP-GOBP domain, which is the typical characteristic of the insect OBPs [27]. Similarly, OBPs have been largely identified in the sequenced *Dipteran* genomes [30]. For example, there are 51 OBPs in *D. melanogaster* and 66 OBPs in *A. gambiae*, respectively.

**Figure 1:**
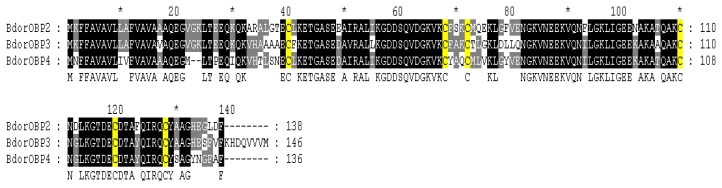
Alignments of the OBP56d group of *B. dorsalis* odorant-binding proteins (OBPs). Amino acid numbering is provided on the right of the alignment. The numbers above the alignment indicate amino acid position in the alignment. All identical and similar amino acid residues are shaded. Yellow boxes show conserved cysteine residues. The letters below the alignment indicate identical residues.

All of the identified BdorOBPs share high sequence homology with their *D. melanogaster* counterparts. The relative identities of BdorOBP7, BdorOBP8, BdorOBP9, and BdorOBP10 compared to DmelOBP83g, DmelOBP83ef, DmelOBP99a, DmelOBP99c were 63%, 47%, 56%, and 54%, respectively. The identities between BdorOBP1, BdorOBP5, BdorOBP6 (cDNA sequence missing 3′ stop code but containing the complete PBP-GOBP domain) and their homologous counterparts of *D. melanogaster* DmelOBP8a, DmelOBP56g, DmelOBP56h, are less than 40%. Notably, BdorOBP2, BdorOBP3 and BdorOBP4 share high identities (83%), and the identities for each pair were 77% (BdorOBP2 and BdorOBP3), 79% (BdorOBP2 and BdorOBP4), 74% (BdorOBP3 and BdorOBP4) (Figure 1). The three OBPs all show the highest identity with DmelOBP56d, with identities of 45%, 47%, 44%, respectively (Table 1). Proteins in the DmelOBP56d group are highly conserved from the signal peptides in the *N*-terminal region to the *C*-terminal region, except for some substitution (Figure 1). Among them, BdorOBP2 has seven additional amino acids in the *C*-terminal region.

These data revealed that the sequence identity between *B. dorsalis* OBPs and their *D. melanogaster* OBPs counterparts varies from 33% to 63%. This is consistent with the level of sequence identity of the previously identified OBPs from other species. The sequence identity between most identified *A. lineolatus* OBPs and their counterparts in other bugs varies from 31% to 53% [27]. Notably, amino acid identity between identified *Culex pipiens quinquefasciatus* OBPs and their mosquito counterparts varies from 17% to 94% [22]. The wide range of amino acid identity could be attributed to the different divergence times between these species (Supplement Figure S1). All of these reports indicate that insect OBPs are divergent proteins among different species.

### 2.2. Homology Alignments of *B. dorsalis* OBPs

**Figure 2:**
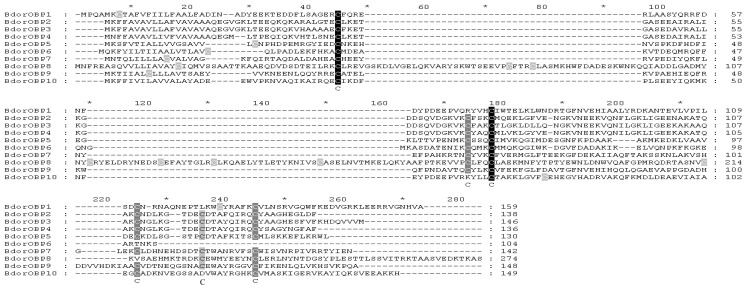
Alignment of the identified *B. dorsalis* OBPs. The classical OBPs: BdorOBP1, BdorOBP2, BdorOBP3, BdorOBP4, BdorOBP5, BdorOBP7, BdorOBP9. “Dimer” OBP: BdorOBP8. The Minus-C OBPs: BdorOBP10. Full-length amino acid sequences of BdorOBPs are aligned by ClustalX 1.83 (Multiple sequence alignment program, Thompson JD, IllkirchCedex, France) and edited using GeneDoc (Sequence analysis program, Karl Nicholas, San Francisco, CA, USA). Accession numbers of the 10 OBPs are listed in Table 1.

The analysis of the derived amino acid sequences showed that they belong to three distinct subgroups: Classical OBPs, Minus-C OBPs and “Dimer” OBPs [7]. The classical OBPs include BdorOBP1, BdorOBP2, BdorOBP3, BdorOBP4, BdorOBP5, BdorOBP7 and BdorOBP9, which have only one six-cysteine motif (Figure 2). BdorOBP10 lacks one cysteine, so belongs to Minus-C OBPs (Figure 2). The BdorOBP6 miss two cysteines due to the partial sequences in the 3′ terminus. BdorOBP8 appears to have one additional six-cysteine motif, and two conserved prolines immediately before the third and the ninth cysteine, respectively, thus belonging to the “Dimer” OBPs (Supplement Figure S2). This is rather similar to some other Dipteran OBPs, including DmelOBP83cd, DmelOBP83ef, GmorOBP3 and GmorOBP7, all of which belong to “Dimer” OBPs group.

### 2.3. Phylogenetic Analysis of Dipteran OBPs

**Figure 3:**
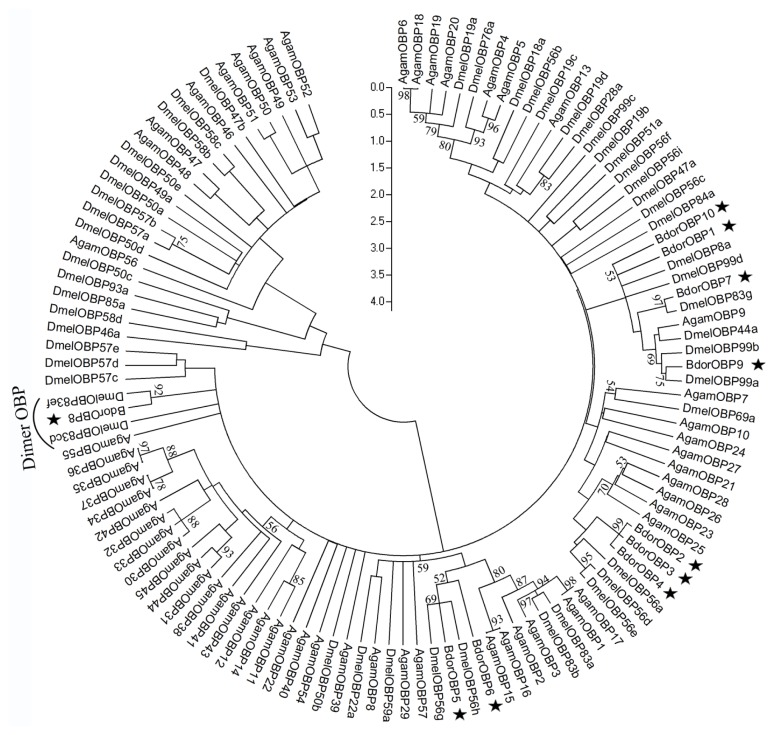
Phylogenetic analysis of BdorOBPs. The sequences used for the analysis included *B. dorsalis* and two other Dipteran species with full genome sequenced: *D. melanogaster* and *A. gambiae*. The highly divergent signal peptide sequences at the *N*-terminus were removed and the neighbor-joining tree was constructed using MEGA 4 with the pair-wise deletion option under the JTT empirical amino acid substitution model. Only bootstrap values above 50 were set to be shown.

A phylogenetic tree was generated to show the relationships among the 10 OBPs of *B. dorsalis* to 108 OBP sequences of other 2 Dipteran species, including *D. melanogaster* and the mosquito *A. gambiae* (Figure 3). These BdorOBPs belong to different groups of orthologous proteins (Figure 3). BdorOBP1, BdorOBP7 and BdorOBP9 are clustered in one group with their *D. melanogaster* counterparts DmelOBP8a, DmelOBP83g, DmelOBP99a, respectively. The other three classic OBPs (BdorOBP2, BdorOBP3 and BdorOBP4) are clustered in a branch with DmelOBP56d, DmelOBP56a and DmelOBP56e, which is consistent with the high amino acid identity observed among them (Table 1). In addition, this phylogenetic analysis supports the high amino acid identity (79%) among these three proteins (BdorOBP2, BdorOBP3 and BdorOBP4). Notably, BdorOBP8 is clustered with other Dipteran “Dimer” OBPs, including DmelOBP83cd and DmelOBP83ef, suggesting that they may have conserved common functions in fly species.

### 2.4. Tissue Expression Pattern of the *OBP* Genes

**Figure 4:**
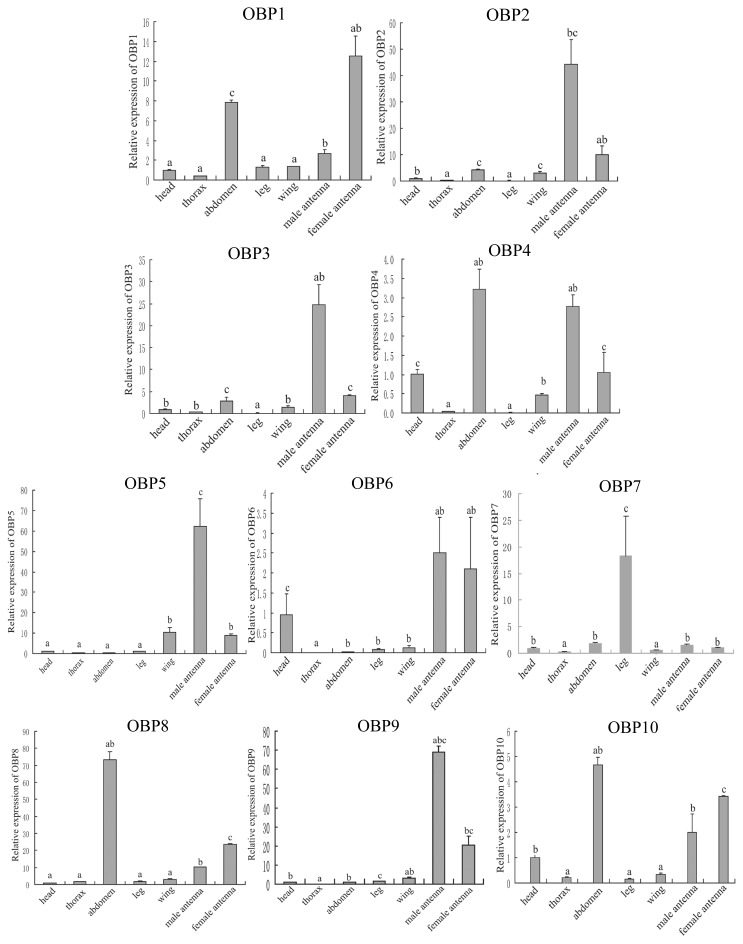
Tissue distribution of the ten identified *OBPs*. qRT-PCR to analyze *OBP* transcript levels in different tissues of mature adults during the oviposition period, including head, thorax, abdomen, leg, wing, male antennae, and female antennae. Relative transcript levels are calculated using β*-actin* as the standard. Different letters indicate significant differences in the expression level of *OBPs* (*p* < 0.05, Duncan’s test). Three biological replicates were performed.

*OBPs* from Tephritid fruit flies have not been largely reported, except some partial sequences of *C. capitata* [28]. We report for the first time the large identification and tissue distribution of *OBPs* in Tephritid fruit flies. qRT-PCR revealed that each of the ten *OBPs* had abundant expression in antenna, except *BdorOBP7*, suggesting that the *B. dorsalis OBP* genes identified in the current study may play an important part in insect olfaction (Figure 4). Among these genes, the expression levels of five classic *OBP* genes (*BdorOBP2*, *BdorOBP3*, *BdorOBP4*, *BdorOBP5*, and *BdorOBP9*) were much higher in male antenna than female antenna, while three *OBP* genes (*BdorOBP1*, *BdorOBP8*, *BdorOBP10*) were expressed much more abundantly in female antenna. The transcript levels of *BdorOBP2*, *BdorOBP3*, *BdorOBP4*, *BdorOBP5*, *BdorOBP9* were 4.4, 6, 2.8, 7.2 and 3.4 times higher in the male antennae, respectively, than in female antennae. There was no significant difference in the expression level of the other two classic *OBPs* (*BdorOBP6* and *BdorOBP7*) in antenna of both sexes.

This result is consistent with previous reports indicating that many olfactory sensory neurons are located in the antennae [31], as is also reported in other insect species. Among 14 identified *AlinOBPs*, 9 *OBP* genes were expressed much higher in both male and female antenna than in other tissues [27]. The transcript levels of *A. funestus* OBPs were much higher in antenna than other tissues [23]. This result is also observed in *Cx. quinquefasciatus*, in which most of the OBPs are mainly expressed in antennae [22]. Three of the *B. dorsalis OBPs* (*BdorOBP1*, *BdorOBP8*, and *BdorOBP10*) have much higher expression in the female antenna than male antenna, revealing that these *OBPs* may be involved in female specific behaviors, such as host location and oviposition. Five *B. dorsalis OBPs* were expressed much more highly in male antenna than female antenna, suggesting that they are possibly necessary for males to be able to mate for courtship.

Additionally, *BdorOBP4* and *BdorOBP10* were also highly present in the heads. Take *BdorOBP4* for example: there was no significant difference between the expression level in the heads and that observed in the female antenna.

Interestingly, four OBPs (*BdorOBP1*, *BdorOBP4*, *BdorOBP8*, and *BdorOBP10*) were also expressed highly in the abdomen, where the reproductive organs are located, indicating that these *OBPs* may have a role in both olfaction and oviposition. The abdomen transcript levels of *BdorOBP8* were much higher than in other tissues. Similarly, abdomen expression was also detected in *Cx. quinquefasciatus* and *A. funestus* [22,23]. Furthermore, it has been reported in two sibling Lepidopteran species *Helicoverpa armigera* and *H. assulta* that *OBP10* is highly abundant in seminal fluid, which is transferred to females during mating and eventually found on the surface of fertilized eggs, suggesting that *OBP10* could be a carrier for oviposition deterrents [32]. Our finding is consistent with the fact that *B. dorsalis* tissues were collected during the oviposition period. Whether or not these four *BdorOBPs* are involved in oviposition deterrence needs further research.

Notably, *BdorOBP7* was specifically expressed in leg, which is another olfactory tissue. Leg expression of *OBPs* has also been reported in other insects, such as *Cx. quinquefasciatus* [22], *A. funestus* [23], *A. lineolatus* [27], and *D. antiqua* [21]. These *OBPs* expressed in the taste sensilla on the legs may be involved in the perception of non-volatile host chemicals and warrant further study.

## 3. Experimental Section

### 3.1. Insect Rearing

The oriental fruit flies were cultured in our laboratory at 28 °C under a photoperiod of 14 h light/10 h dark. Adult flies were reared on artificial diets described previously, and hatched larvae were maintained in bananas [33].

### 3.2. Identification of the *OBP* Genes and Sequence Analysis

*OBP* genes were first identified using BLASTN and BLASTX results from our *B. dorsalis* cDNA library [29], which was established from three samples: larvae of three instars, pupae from different stages, newly emerged adults and sex matured adults before and after copulation (the sex ratio was 1:1). At least ten insects were collected for each stage. The 10 ESTs showing homology to odorant-binding protein (OBP) family genes were compared to one another and the sequences that include the complete opening reading frame, several conserved cysteines, and the signal peptides in *N* terminus were chosen for further study. Homology analysis was performed with BLASTX [34]. The *OBP* genes were translated, and the characteristics of the obtained proteins were predicted using the Expert Protein Analysis System [35]. Sequence alignment was conducted using the ClustalW and GENDOC computer programs [36,37]. The putative *N*-terminal signal peptides were predicted by Simple Modular Architecture Research Tool [38].

### 3.3. Phylogenetic Analysis

Amino acid sequences of ten putative *OBP* genes identified in this study along with the *OBPs* from two other Dipteran species (*D. melanogaster* and *A. gambiae*) were used to construct a phylogenetic tree using MEGA4.0 (Molecular Evolutionary Genetics Analysis, Version 4.0, Sudhir Kumar, AZ, USA) with the pair-wise deletion option under the JTT empirical amino acid substitution model [30]. The highly divergent signal peptides in the *N* terminal were removed. Branch support was assessed by bootstrap analysis with 1000 replicates. A species phylogenetic tree was also generated using Interactive Tree Of Life (iTOL) web tool [39] (http://itol.embl.de) to show the relationships between *B. dorsalis* and other mentioned species, including *C. capitata*, *D. melanogaster*, *Glossina morsitans morsitans*, *D. antiqua*, *A. gambiae*, *A. funestus*, *Aedes albopictus*, and *Cx. quinquefasciatus*.

### 3.4. Total RNA Isolation and cDNA Synthesis

Total RNA was extracted from different tissues of the mature adult during the oviposition period, including head, thorax, abdomen, leg, wing, male antennae, and female antennae, according to methods previously described by Zheng *et al*. [40]. Matings were obtained by placing 100 mature males and 100 mature females (two weeks old) into 60 cm × 60 cm × 60 cm boxes (20 flies for each sex per box) 2 h before dusk. The boxes were then checked 1 h later under dim light, and mating pairs were gently coaxed into another new boxes. The following morning, all unmated oriental fruit flies were discarded. The tissue samples were dissected from 40 individuals of each sex. Two micrograms of total RNA was reverse transcribed into cDNA using M-MLV Reverse Transcriptase (FirstStrand cDNA Synthesis Kit, Takara, Japan) with the primer oligo-anchor R (5′-GACCACGCGTATCGATGTCGACT_16_ (A/C/G)-3′). The integrity of the total RNA was examined using 1% agarose electrophoresis, and the purity was determined by the ratio of A260/A280 measured by a spectrophotometer. To eliminate contamination from genomic DNA, a genomic DNA elimination column that could efficiently remove genomic DNA was used after total RNA extraction.

### 3.5. Expression Pattern Analysis of *OBPs* by Quantitative Real-time PCR

The gene-specific primers (Table 2) were used to conduct qRT-PCR to detect the tissue distribution of *OBPs* using a SYBR Premix ExTaq kit (Takara, Dalian, China) following the manufacturer’s instructions with a real-time thermal cycler (Bio-Rad, Hercules, CA, USA). β*-Actin* was amplified for internal standardization. The PCR efficiency of the genes was validated before gene expression analysis. The qRT-PCR was performed in a volume of 20 μL containing 10 μL of 2× SYBR Premix ExTaq™, 2 μL cDNA (1:100 diluted), 2 μL each of forward and reverse primers (1 μmol/L), and 2 μL nuclease free H_2_O. The qRT-PCR program was as follows: 94 °C for 3 min followed by 40 cycles at 94 °C for 15 s, 60 °C for 30 s, and 72 °C for 20 s. At the end of each PCR reaction, the melting curve was analyzed for the PCR products to confirm the presence of a single fragment of amplification. The expression level of each *OBP* was then calculated by 2^−ΔCT^ using the comparative CT method in which the discrepancy between the CT for the *OBP* and β*-actin* (ΔCT) was calculated to normalize the variation in the amount of cDNA in each reaction. Three biological replicates were performed.

### 3.6. Statistical Analysis

All the results from experimental replicates were analyzed by one-way analysis of variance (ANOVA) and Duncan’s test using SPSS 16.0 (IBM Corporation, Somers, NY, USA).

## 4. Conclusions

This work presents for the first time a study on the *OBPs* in Tephritid fruit flies, which are of major economic importance in agriculture. Despite some advances during the past few decades, no new approaches to Tephritid fruit flies control have been widely and effectively used. Transcriptome sequencing has provided insights into an approach to identify functional genes in important biological processes, such as development and reproduction [29,41]. In our study, 10 *OBP* genes were identified from *B. dorsalis* for the first time. Tissue distribution of the *OBP* genes showed that most of them are highly expressed in the antenna of both sexes. *BdorOBP7* is specifically expressed in leg, and four *OBPs* (*BdorOBP1*, *BdorOBP4*, *BdorOBP8*, and *BdorOBP10*) are also enriched in the abdomen, indicating that they may function in other biological processes. Large-scale functional and structural characterization of these *OBPs* will be the objectives of our further research.

## Supplementary Information



## Figures and Tables

**Table 1:** Oriental fruit fly assembled sequences with best-hit matches to *D. melanogaster* odorant binding protein genes.

Gene name	Accession number	Length	*Drosophila* gene	Subject ID	*E*-value	Identity (%)
*BdorOBP1*	KC559112	159	OBP8a	ACY92783	1 × 10^−14^	33
*BdorOBP2*	KC559113	138	OBP56d	NP_611444	2 × 10^−21^	45
*BdorOBP3*	KC559114	146	OBP56d	NP_611444	8 × 10^−22^	47
*BdorOBP4*	KC559115	136	OBP56d	NP_611444	1 ×10^−19^	44
*BdorOBP5*	KC559116	130	OBP56g	NP_611447	3 ×10^−14^	34
*BdorOBP6*	KC559117	104 *	OBP56h	ABW78079	1 × 10^−11^	36
*BdorOBP7*	KC559118	142	OBP83g	AAN13232	2 × 10^−54^	63
*BdorOBP8*	KC559119	274	OBP83ef	AAF51918	1 × 10^−69^	47
*BdorOBP9*	KC559120	148	OBP99a	ACT22143	2 × 10^−56^	56
*BdorOBP10*	KC559121	149	OBP99c	ACT22281	3 × 10^−46^	54

Length, number of amino acids including signal peptide region;

* partial sequence in the 3′ terminal.

**Table 2:** Primers used for gene expression detection of *BdorOBPs* by qRT-PCR.

Primer name	(5′→3′) Nucleotide sequence
BdorOBP1RTF	GGCGAGCGTTGTTTCCAG
BdorOBP1RTR	ATCGGCACCAGCACTTCC
BdorOBP2RTF	TTCTTCGCTGTTGCTGTTTTGC
BdorOBP2RTR	AGAAGCATTTGACTTTGCCATC
BdorOBP3RTF	TTGACCGAGGAGCAGAAAC
BdorOBP3RTR	TTGACTTTGCCATCGACTT
BdorOBP4RTF	GAACTTCTTCGCTGTTGCTG
BdorOBP4RTR	CTTGACTGTCATCGCCTTTG
BdorOBP5RTF	CCACGACTTCATTGAGGGTA
BdorOBP5RTR	CCACAGCAGCAACTAATTTATC
BdorOBP6RTF	ACGAAGCCAAAGTCACGG
BdorOBP6RTR	GCATCAAAGACGCCATCC
BdorOBP7RTF	GTTGCAGGCAAATTCCAGAT
BdorOBP7RTR	AGAGTCCCATTCGCTCCACA
BdorOBP8RTF	CGAAGTCGGCAGCAAAGA
BdorOBP8RTR	TCGTCCGCATCGAACCAG
BdorOBP9RTF	GCGATGCCGACCATGATGAC
BdorOBP9RTR	ACCACCACGATAAGCCCACT
BdorOBP10RTF	GTGTCTTCTGCGAACATGAGG
BdorOBP10RTR	CACTTGTGACCACGATAGGC
β-actinRTF	CTCGTCCAACCGTTCATACC
β-actinRTR	CTGACCTGCCCACTGAAGTT
