# Supplementary material for: Identification and Expression Profile Analysis of Odorant Binding Proteins in the Oriental Fruit Fly Bactrocera dorsalis
Source: Int J Mol Sci. 2013 Jul 17;14(7):14936–49. doi: 10.3390/ijms140714936 (PMC3742281; doi:10.3390/ijms140714936)

Supplementary Information

Figure S1. A species phylogenetic tree to show the relationships between different species, including *Anopheles gambiae*, *Anopheles funestus*, *Aedes albopictus*, *Culex quinquefasciatus*, *Drosophila melanogaster*, *Bactrocera dorsalis*, *Delia antiqua*, *Ceratitis capitata*, and *Glossina morsitans morsitans*.

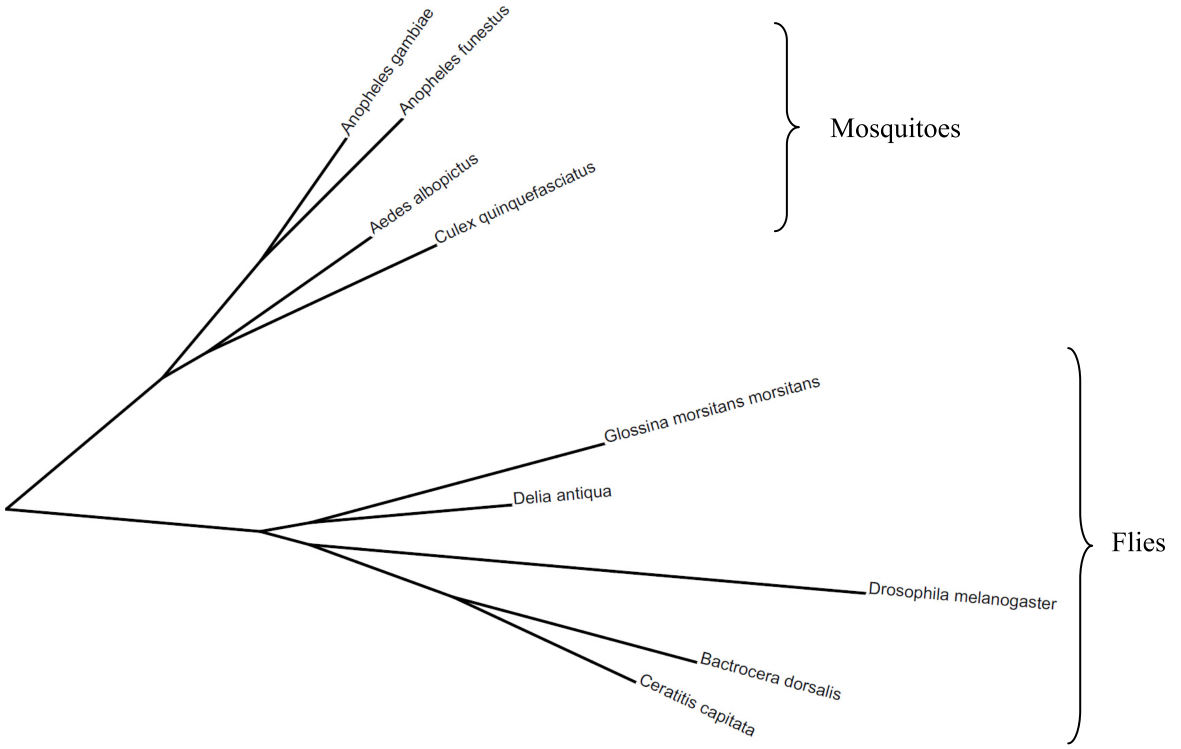

Figure S2. Alignment of the "Dimer" OBP (BdorOBP8) with those "Dimer" OBPs of other insect species. Full-length amino acid sequences of BdorOBP8 are aligned by ClustalX 1.83 and edited using GeneDoc. Yellow boxes show the conserved cysteine and black boxes show proline residues in the "Dimer" OBPs. The other insect species are: *D. melanogaster* (Dmel) and *G. morsitans morsitans* (Gmor). GenBank accession numbers for the "Dimer" OBPs are: Dmel OBP83ef: NP_731042; Dmel OBP83cd: NP_649612; GmorOBP3: CBA11307; GmorOBP7: CBA11311.

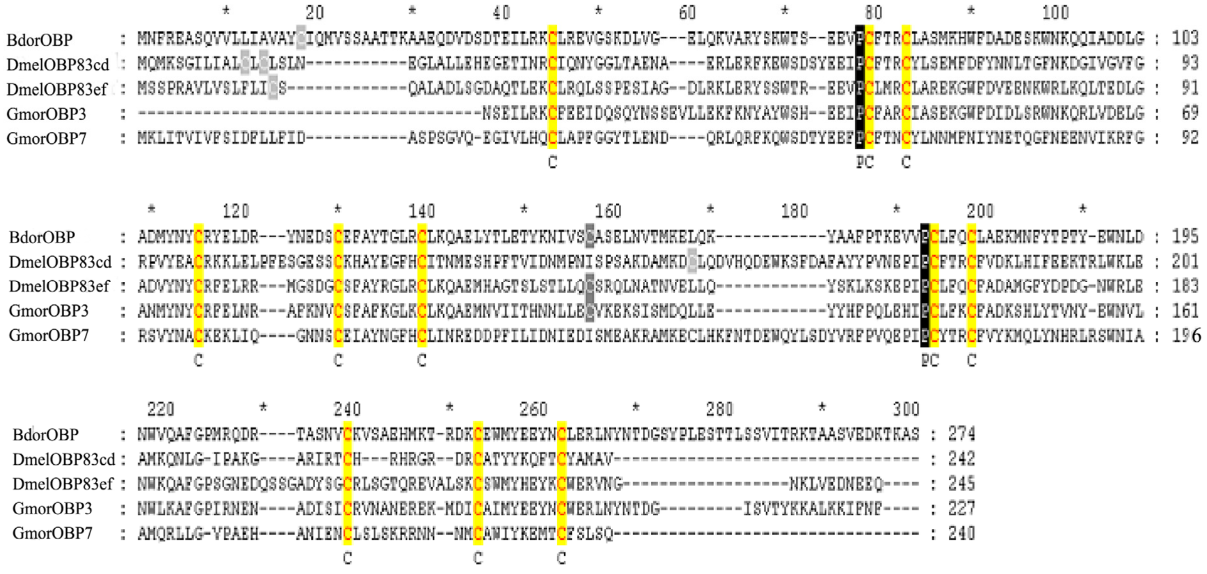

Supplement: Supplementary file 1 [file ijms-14-14936-s001.pdf]
